# Hepatic sclerosing haemangioma showing restricted diffusion: A case report with histopathologic correlation

**DOI:** 10.1259/bjrcr.20220029

**Published:** 2022-05-12

**Authors:** Shintaro Takinoshita, Hideki Ishimaru, Shinji Okano, Tetsuhiro Otsuka, Yutaka Ishimaru, Shuhei Miyazaki, Jun Nakagawa, Miyuki Koga, Akihiko Soyama, Masaaki Hidaka, Masayuki Fukumoto, Susumu Eguchi, Masataka Uetani

**Affiliations:** 1 Department of Radiology, Nagasaki University Hospital, Nagasaki, Japan; 2 Department of Radiology, Sasebo Chuo Hospital, Sasebo, Japan; 3 Department of Pathology, Nagasaki University Hospital, Nagasaki, Japan; 4 Department of Radiology, Nagasaki Prefecture Shimabara Hospital, Shimabara, Japan; 5 Department of Surgery, Nagasaki University Graduate School of Biomedical Sciences, Nagasaki, Japan

## Abstract

Hepatic sclerosing haemangiomas are rare benign tumours that are often difficult to distinguish from malignant tumours because these tumours do not show the typical imaging features of cavernous haemangiomas. We report a case of a sclerosing haemangioma that showed restricted diffusion and was difficult to differentiate from a malignancy.

A 60-year-old female was referred to our hospital for evaluation of a hepatic mass that was incidentally diagnosed after a CT scan for right lower quadrant abdominal pain.

Contrast-enhanced dynamic CT showed hepatic capsular retraction, with a small peripheral enhancement of the mass. The lesion appeared homogeneously hypointense on *T1W* images, heterogeneously hyperintense on *T2W* images, hyperintense on diffusion-weighted images, and hypointense on apparent diffusion coefficient (ADC) map. The lesion was suspected to be a cholangiocellular carcinoma and was surgically resected, but a final diagnosis of hepatic sclerosing haemangioma was made.

Hepatic sclerosing/sclerosed haemangiomas are usually considered to show an increased ADC, which is useful for distinguishing them from malignant tumours. However, in this particular case, most of the lesion contained many obliterated or narrowed vascular channels, which might have acted as septa restricting the diffusion of water molecules in the intervening fibrous and/or hyalinised tissue. Hepatic sclerosing haemangiomas in the process of becoming completely fibrotic may show restricted diffusion, similar to malignant tumours.

## Introduction

Hepatic cavernous haemangiomas are the most common noncystic hepatic lesions, accounting for 20% of benign hepatic tumours^1^ and are composed of many dilated and thin-walled vessels filled with erythrocytes. Typical hepatic haemangiomas demonstrate nodular peripheral enhancement in the arterial phase and an increase in centripetal filling in the delayed phase on contrast-enhanced dynamic computed tomography (CT) or magnetic resonance imaging (MRI).^2^

Hepatic sclerosing/sclerosed haemangiomas are relatively rare benign tumours that are believed to result from regressive changes secondary to thrombosis, infarction or haemorrhage in cavernous haemangiomas.^3^ It is often difficult to distinguish hepatic sclerosing/sclerosed haemangiomas from malignant tumours because these tumours do not show the typical imaging features of cavernous haemangiomas. We report a case of a sclerosing haemangioma that showed restricted diffusion and was difficult to differentiate from a malignancy.

## Case report

A 60-year-old female was referred to our hospital for evaluation of a hepatic mass, which was incidentally diagnosed when the patient underwent a CT scan for right lower quadrant abdominal pain. She had no history of hepatic disease or other malignancies. Her laboratory examinations revealed no increases in the levels of the evaluated tumour markers, including α-fetoprotein, carcinoembryonic antigen and carbohydrate antigens 19–9 and 15–3.

**Figure 1.**
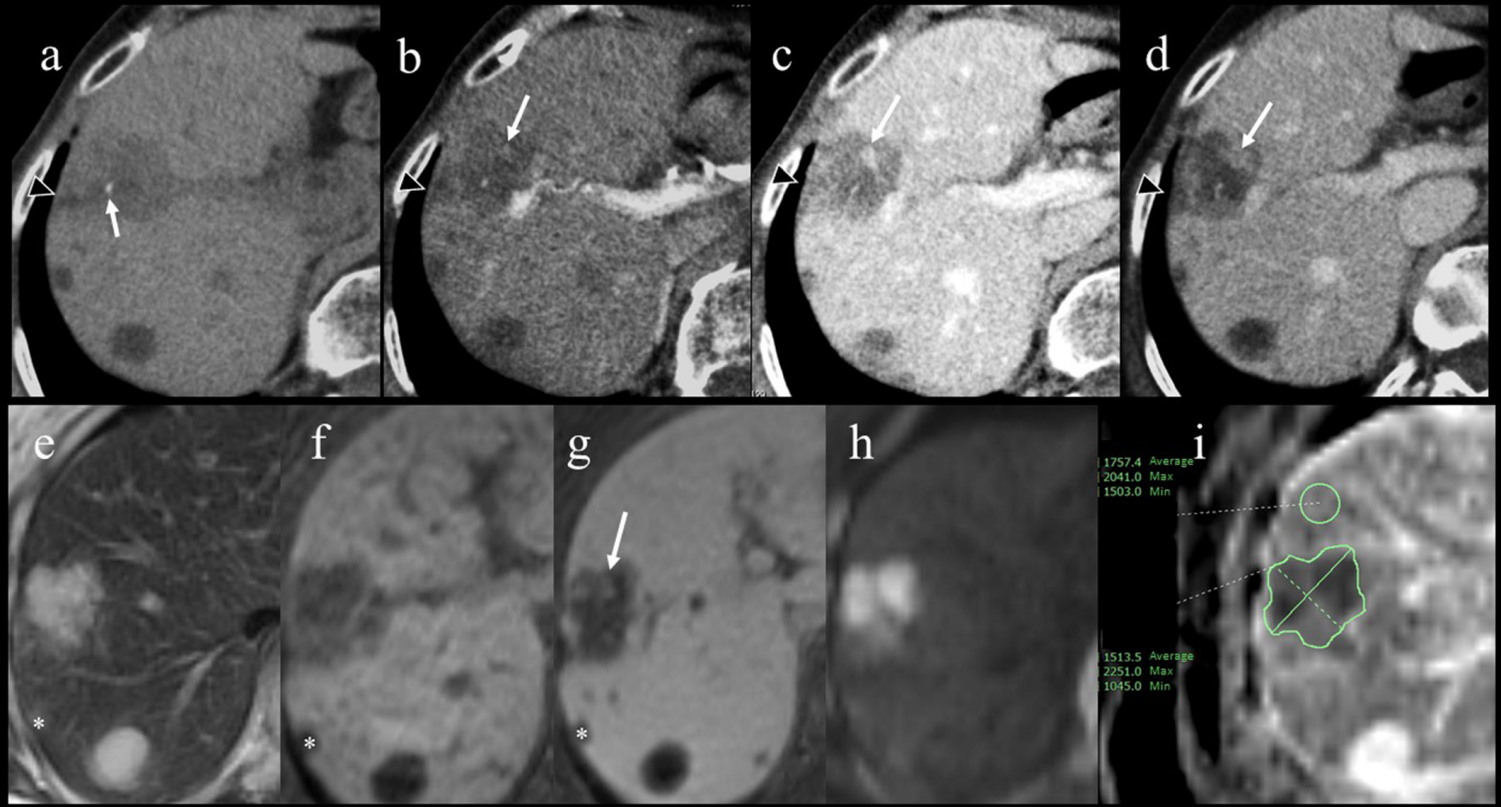
Precontrast CT (**a**) showed a low-attenuated irregular mass on the S8 surface and a small amount of internal calcification (arrow). Contrast-enhanced dynamic CT (**b-d**) showed a small nodular edge enhancement during the arterial phase (**b**) that spread around the nodule during the portal phase (**c**) and lasted until the delayed phase (**d**) in the ventral medial portion of the tumour (arrow), but no enhancement in other areas. Capsular retraction adjacent to the lesion was also noted (arrowhead). The lesion demonstrated heterogeneous hyperintensity on T2WI (**e**), homogeneous hypointensity on fat-suppressed gradient-echo T1WI (**f**), and a small area of enhancement in the ventral portion (arrow) on the equilibrium phase of contrast-enhanced dynamic fat-suppressed gradient-echo T1WI (**g**). The lesion showed hyperintensity on DWI (**h**) and hypointensity on ADC map (**i**), suggesting diffusion restriction. The cyst on the liver surface (asterisks) was used as a landmark in the radiologic-histologic correlation.

Precontrast CT showed an irregular mass that was 35 mm in size with low attenuation in the right anterosuperior area (segment 8) of the liver; additionally, spotty calcification was observed inside the mass. The lesion exhibited a small nodular edge enhancement during the arterial phase that spread around the nodule during the portal phase and lasted until the delayed phase, as well as extensive internal unenhanced areas, on contrast-enhanced dynamic CT (Figure 1a–d). Hepatic capsular retraction adjacent to the lesion was also noted. Hepatic sclerosing haemangioma and cholangiocellular carcinoma were included in the differential diagnosis, and MRI was performed to further investigate the characteristics of the lesion.

The mass appeared homogeneously hypointense on *T1W* images (T1WI) and heterogeneously hyperintense on *T2W* images (T2WI) relative to the hepatic parenchyma. Gadoxetic acid–enhanced dynamic MRI was performed, but severe motion-related artefacts due to breath-holding failure degraded the images of the arterial and portal phases. The enhancement pattern that was observed on dynamic CT was also observed in the equilibrium phase of contrast-enhanced dynamic fat-suppressed gradient-echo T1WI (Figure 1g–h).

The lesion showed hyperintensity on diffusion-weighted imaging (DWI), with a b value of 800, and hypointensity on the apparent diffusion coefficient (ADC) map, which suggested diffusion restriction (Figure 1j and k). When measured with a region of interest surrounding the entire lesion, the mean ADC of the lesion was 1.51 × 10^−3^ mm^2^/s (range: 1.04–2.25 × 10^−3^ mm^2^/s). On the other hand, the mean ADC of the background liver close to the lesion was 1.76 × 10^−3^ mm^2^/s (range: 1.50–2.04 × 10^−3^ mm^2^/s) (Figure 1i).

**Figure 2.**
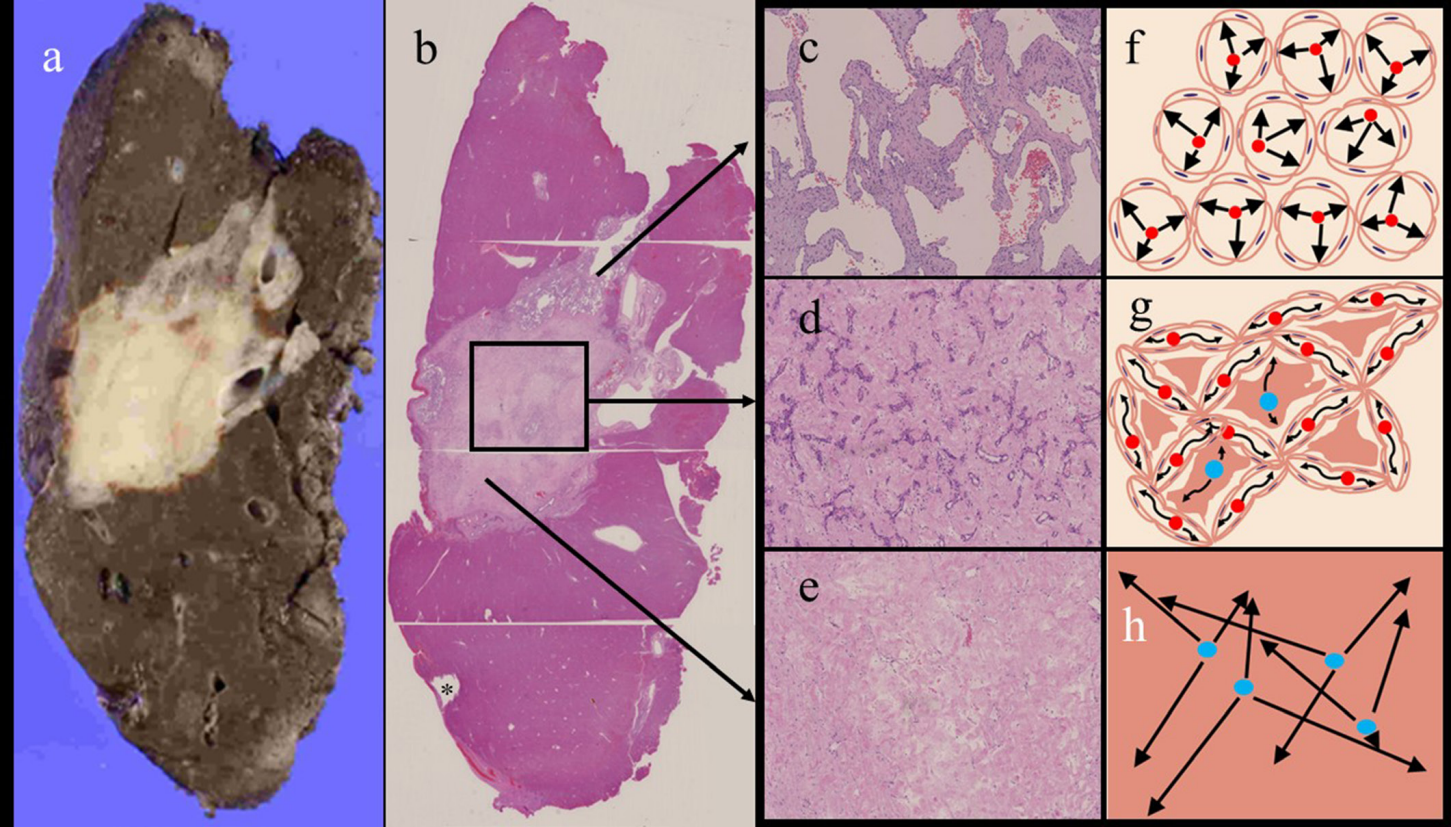
The gross appearance of the cut surface of the hepatic mass (**a**) and the microscopic findings on haematoxylin and eosin (H&E) staining (**b-e**). The cyst on the liver surface (asterisk) was used as a landmark in the radiologic-histologic correlation (**b**). There was a small lesion showing typical features of cavernous haemangioma, with variably sized vascular spaces lined by a single layer of flat endothelial cells (H&E x100) (**c**). The tumour was largely composed of obliterated or narrowed vascular channels and surrounding sclerotic and/or hyalinised tissue (**d**). Some hyalinised/fibrous areas with poorly cellular components were also observed in the small part of the mass (**e**). This hyalinisation was accompanied by the presence of liquiform degeneration. Schematic drawings (**f-h**) show the movement of water molecules within the typical cavernous haemangioma (**f**), the degenerated cavernous haemangioma containing obliterated or narrow vascular channels surrounding sclerosed and/or hyalinised tissue (**g**), and hyalinised but poorly cellular tissue (**h**).

Cholangiocellular carcinoma was suspected based on the restricted diffusion, and partial hepatectomy was performed. On gross pathology, the mass was a well-demarcated hepatic mass that was composed of two lesions: one was a homogeneous white lesion, and the other was a red–brown lesion with a honeycomb-like appearance, which was observed in a small peripheral area of the mass (Figure 2a). The microscopic examination revealed that the white lesion consisted of abundant hyalinised stroma containing many narrow or collapsed vascular lumina that were lined by endothelial cells (Figure 2d). Some hyalinised areas with poorly cellular but fibrous components were also observed in the white lesion (Figure 2e). On the other hand, the red–brown lesion was revealed to be a typical cavernous haemangioma with many variably sized vascular spaces, which were lined by a single layer of flat endothelial cells and containing some red blood cells (Figure 2c). The final diagnosis was sclerosing haemangioma. The postoperative course was uneventful, and no recurrence developed within 2 years after surgery.

## Discussion

Hepatic sclerosed/sclerosing haemangioma is a rare type of hepatic haemangioma, and its imaging appearance is similar to that of malignant hepatic tumours, such as cholangiocellular carcinoma and hepatic metastases, which could result in misdiagnosis and unnecessary surgical resection.^4–6^ Hepatic sclerosed/sclerosing haemangiomas exhibit irregular shapes and heterogeneous densities or signal intensities, including slightly low densities on CT, slightly low signal intensities on T1WI, and lower signal intensities on T2WI than typical cavernous haemangiomas.^2^ Hepatic sclerosed haemangiomas exhibit little or no enhancement during the arterial phase, only subtle linear marginal enhancement during the delayed phase, or peripheral heterogeneous enhancement, with most lesions showing no enhancement. On the other hand, hepatic sclerosing haemangiomas exhibit a trend of centripetal enhancement characteristics, as well as extensive internal unenhanced areas.^2^ A lower signal intensity than cerebrospinal fluid on T2WI and lack of enhancement on dynamic contrast imaging also suggests the possibility of malignant tumours.^4^ DWI can help to characterise and distinguish benign from malignant lesions based on ADC measurements.^7^ Malignant lesions show low ADC values, likely due to their high cellularity and the resultant restricted diffusion of water molecules. In contrast, cavernous haemangiomas and cysts have higher ADC values than other malignant lesions due to their higher fluid content, which results in more freedom for water molecule diffusion.^7^ There have been a few reports on the DWI and ADC values of hepatic sclerosing/sclerosed haemangiomas. In a previous study, the median ADC value of 18 sclerosing haemangiomas was reported to be significantly higher than that of hepatic malignancies (1.852 × 10^−3^ mm^2^/s *vs* 1.005 × 10^−3^ mm^2^/s).^8^ According to Miyata et al, the average ADC value of five hepatic sclerosed haemangiomas was 1.94 × 10^−3^ mm^2^/s (range: 1.73–2.10 × 10^−3^ mm^2^/s), which was higher than the values of common malignant liver tumours.^6^ According to another paper by Jia et al, the mean ADC value of 12 hepatic sclerosed haemangiomas was 1.684  ±  0.476 × 10^–3^ mm^2^/s, which was higher than that of the surrounding liver parenchyma.^2^ These three papers stated that ADC values may be useful for distinguishing sclerosing/sclerosed haemangiomas from malignant tumours.

In our case, the mean ADC of the mass was 1.51 × 10^−3^ mm^2^/s. Furthermore, the lesion showed higher intensity on DWI and lower intensity on the ADC map than the normal liver parenchyma. Although there is significant variability in ADC values depending on the coil system, imagers, vendors, and field strengths that are used for MRI,^9^ there is no doubt that the hepatic sclerosing haemangioma in our case showed restricted diffusion.

Cavernous haemangiomas consist of cavernous vascular spaces that allow for the free diffusion of water molecules and intervening interstitial septa that restrict molecular motion.^10^ Haemangiomas with wider cavernous spaces allow for more freedom in terms of molecular motion (Figure 2f). Conversely, haemangiomas with narrower cavernous spaces have more abundant and thicker irregular intervening septa, which could disturb molecular diffusion, resulting in lower ADC values.^10^ The increased ADC value of a sclerosed haemangioma can be explained by the presence of many hyalinised portions with poorly cellular but fibrous components accompanied by liquiform degeneration (Figure 2h).^5^

In our case, large parts of the tumour contained many obliterated cavernous spaces surrounding fibrous and/or hyalinised tissue. The obliterated cavernous spaces may act as septa, which could restrict the diffusion of water molecules in the intervening fibrous and/or hyalinised tissue (Figure 2g) and result in a decreased ADC value.

The sclerosing haemangioma in our case had a low signal on ADC map overall but showed some heterogeneity. The slightly hyperintense areas on the ADC map may have reflected the presence of small typical cavernous haemangiomas or small areas of pure hyalinised stroma. However, due to the low spatial resolution (thickness, 5 mm) of MRI and the inconsistency with the sections of the excised specimen, it was not possible to assess the ADC values in the small amount of cavernous haemangioma tissue or the poorly cellular hyalinised area. Despite the ambiguity described above, it is certain that the restricted diffusion of the main component of the mass was due to the obliterated cavernous spaces surrounding the fibrous and/or hyalinised tissue. These histopathological features may be due to the process of fibrosis.

## Conclusion

Radiologists should be aware that hepatic sclerosing haemangiomas in the process becoming completely fibrotic can show restricted diffusion and masquerade as malignant tumours.

## Learning points

- Sclerosing haemangiomas are the result of the degeneration of cavernous haemangiomas due to haemorrhage or infarction and show similar imaging findings to malignancies such as cholangiocellular carcinoma.
- The ADC values of sclerosing haemangiomas are generally considered to be lower than those of malignant tumours and are useful for distinguishing them from malignant tumours.
- Exceptionally, sclerosing haemangiomas in the process becoming completely fibrotic may show low ADC values similar to those of malignant tumours because obliterated vascular channels that are lined by endothelial cells may act as septa and restrict the diffusion of water molecules in the intervening hyalinised tissue.
